# Supplementary material for: Development of a Potency Assay for Nous-209, a Multivalent Neoantigens-Based Genetic Cancer Vaccine
Source: Vaccines (Basel). 2024 Mar 19;12(3):325. doi: 10.3390/vaccines12030325 (PMC10975996; doi:10.3390/vaccines12030325)
Supplement: Supplementary file 1 [file vaccines-12-00325-s001.zip › vaccines-2890390-supplementary.pdf]

Table S1. Sequence of oligoes and probes used for FSP transgene specific or house-keeping Q-PCRs.

**Table S1.** Sequence of oligoes and probes used for FSP transgene specific or House-keeping Q-PCRs			
**Target sequence**	**Forward**	**Dual-labeled Probe**	**Reverse**
FSP-A1	AAGAAACGGCGGAAAGAAAT	5' FAM-CTATGAGCCTGGCCTGCAG-3' Tamra	CCACAAAAGACACGGTGATG
FSP-A2	GCATCCAGAAGAGAGCCATC	5' FAM-CCGTGCTGAAACTGCTGAAT-3' Tamra	GTGTTCTCGAGAGGCAGGTC
FSP-A3	GAGAAGAAACGGCCTGAGTG	5' FAM-AGGGACACACAAAGAATCGG-3' Tamra	TCTTCTTGTGCACGATCTGG
FSP-A4	CTGAGGCCACACAGAGTTCA	5' FAM-CTCCTGCTGGCCAGAGACT-3' Tamra	CAGCAGTTGGAAGCCCTTAG
FSP-B1	GGAGATCTTCCGGATTCACA	5' FAM-ATGGCGCTAGGCATAGCAGA-3' Tamra	GACCAGGACCTTGGAGAACA
FSP-B2	TGCAAAACTTCGCCTTCTTCA	5' FAM-AACAAGTACATCAATATCCTGACGCGGCG-3' Tamra	CCGCAGTGGTCCTCTCTTTTC
FSP-B3	CACTCAGGCCGTTTCTTCTC	5' FAM-AACAGCAGCAGAGGGTGTCT-3' Tamra	CCTGCCTATTCCTCCACAC
FSP-B4	GGTGTCCGCCGATTTCAG	5' FAM-CCATCTGCTGATCAACGAGGGCAA-3' Tamra	CAACCGGTAGGCATTTCCA
human β-Actin	ATGATGATATCGCCGCGCT	5' VIC-CGTCTTCCCCTCCATCGTG-3' Tamra	GAATCCTTCTGACCCATGCCC
human HPRT	TGCTGAGGATTTGGAAAGGGT	5' VIC-ACTGAACGTCTTGCTCGAGA-3' Tamra	GGCCTCCCATCTCCTTCATC

Table S2. Amount of each DS present in two clinical DP lots as quantified by transgene specific Q-PCRs

Table S2a. Amount of each DS present in two GAd-209-FSP clinical DP lots as quantified by transgene specific Q-PCRs

GAd-209-FSP	vp/ml (Lot #RL18-0010)	vp/ml (Lot #RL22-0026)
GAd-209-FSP-A1	5.72×10^{10}	7.82×10^{10}
GAd-209-FSP-A2	6.73×10^{10}	5.73×10^{10}
GAd-209-FSP-A3	6.25×10^{10}	7.7×10^{10}
GAd-209-FSP-A4	6.44×10^{10}	7.48×10^{10}

Table S2b. Amount of each DS present in two MVA-209-FSP clinical DP lots as quantified by transgene specific Q-PCRs

MVA-209-FSP	vp/ml (Lot #RL19-0002)	vp/ml (Lot #RL21-0005)
MVA-209-FSP-B1	2.45×10^{9}	3.96×10^{9}
MVA-209-FSP-B2	1.09×10^{9}	1.07×10^{9}
MVA-209-FSP-B3	6.78×10^{8}	9.44×10^{8}
MVA-209-FSP-B4	1.43×10^{9}	1.43×10^{9}

Table S3. Specificity of FSPs transgenes-specific Q-PCR assays.

**Table S3a.** Specificity of FSP-A transgene specific Q-PCR assays					
**Vector**	**Titer with Universal qPCR**	**Titer with FSP-A1 qPCR**	**Titer with FSP-A2 qPCR**	**Titer with FSP-A3 qPCR**	**Titer with FSP-A4 qPCR**
GAd-209-FSP-A1	3.5×10^{11}	5.2×10^{11}	nd	nd	nd
GAd-209-FSP-A2	4×10^{11}	nd	3.8×10^{11}	nd	nd
GAd-209-FSP-A3	4.75×10^{11}	nd	nd	5.2×10^{11}	nd
GAd-209-FSP-A4	3.5×10^{11}	nd	nd	nd	3.9×10^{11}

**Table S3b.** Specificity of FSP-B transgene specific Q-PCR assays					
**Vector**	**Expected Amount**	**Titer with FSP-B1 qPCR**	**Titer with FSP-B2 qPCR**	**Titer with FSP-B3 qPCR**	**Titer with FSP-B4 qPCR**
pFSP-B1	5×10^{7}	5.7×10^{7}	27	0.7	3
pFSP-B2	5×10^{7}	2.6	5.4×10^{7}	nd	nd
pFSP-B3	5×10^{7}	8.6	nd	5.7×10^{7}	26
pFSP-B4	5×10^{7}	nd	nd	3.7	4.8×10^{7}

Table S4. Quality attributes of GAd and MVA vectors.

Table S4a. Physical titer (vp/ml), infectious titer (ifu/ml) and vp/ifu ratio of two GAd-209-FSP clinical DP lots

**GAd-209-FSP**	**vp/ml (Lot #RL18-0010)**	**vp/ml (Lot #RL22-0026)**
vp/ml	1.88×10^{11}	2.48×10^{11}
ifu/ml	1.88×10^{9}	3.11×10^{9}
vp/ifu ratio	100	80

Table S4b. Physical titer (vp/ml), infectious titer (ifu/ml) and vp/ifu ratio of two MVA-209-FSP clinical DP lots

**MVA-209-FSP**	**vp/ml (Lot #RL19-0002)**	**vp/ml (Lot #RL21-0005)**
vp/ml	3.4×10^{9}	4.33×10^{9}
ifu/ml	1.65×10^{8}	1.96×10^{8}
vp/ifu ratio	20.6	22.1

Figure S1

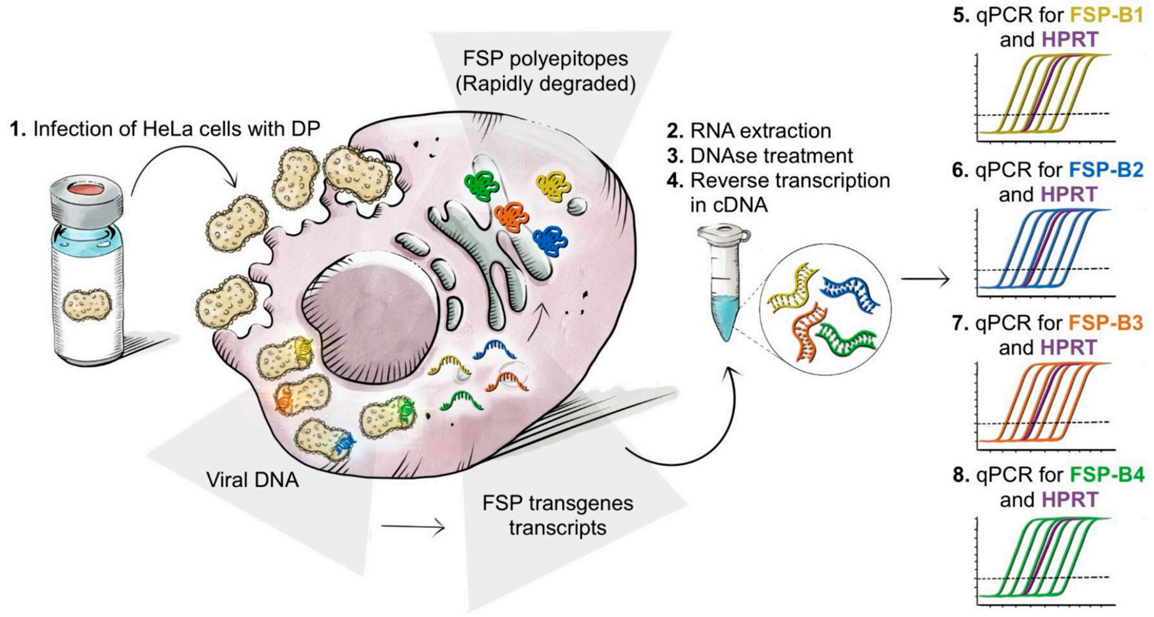

Figure S1: Schematic representation of the RT-Q-PCR transgene expression potency assay for MVA-209-FSP testing. HeLa cells are infected with MVA-209-FSP DP (1), containing four different vectors as depicted. Total RNA is extracted from infected cells (2), treated with DNAse to eliminate vector genome (3) and reverse transcribed to cDNA (4). Transcript levels for each of the four FSP-B transgenes are assessed by four independent RT-Q-PCRs (5 to 8) with transgene specific oligo/probe/standard curve sets, indicated by the corresponding colors (FSP-B1 yellow, FSP-B2 blue, FSP-B3 orange, FSP-B4 green). Each Q-PCR assay is a duplex reaction in which, simultaneously to the absolute quantification of the target FSP-B transgene transcript, the HPRT transcript is also amplified with specific oligo/probes (depicted in purple). The latter is used as housekeeping internal control for RNA integrity and reverse transcription efficiency based on the obtained Cq value.

Figure S2

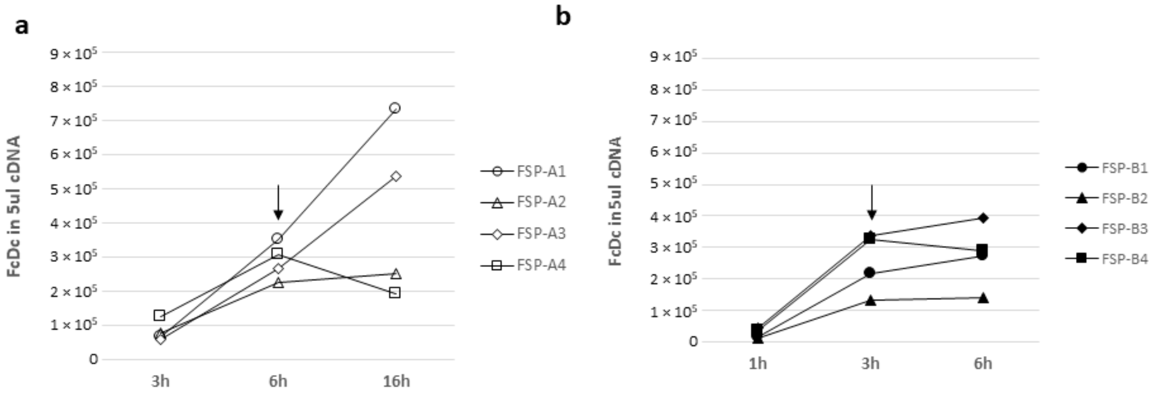

Figure S2: Time course analysis of FSP transcript levels in HeLa cells infected with GAd-209-FSP or MVA-209-FSP vectors. a) HeLa cells were infected at MOI 80 with GAd-209-FSP DP. RNA from infected cells was extracted at the indicated time point post infection and reverse transcribed to cDNA. Transcript levels for each of the four FSP-A transgenes at each time point were assessed by RT-Q-PCR with transgene specific oligo/probe/standard curve sets. The number of copies for each transcript (FcDc= FSP cDNA copies) detected in 5µl of cDNA is shown. b) HeLa cells were infected at MOI 0.3 with MVA-209-FSP DP. RNA from infected cells was extracted at the indicated time point post infection and reverse transcribed to cDNA. Transcript levels for each of the four FSP-B transgenes at each time point were assessed by RT-Q-PCR with transgene specific oligo/probe/standard curve sets. The number of copies for each transcript (FcDc= FSP cDNA copies) detected in 5µl of cDNA is shown. Arrows indicate the time points selected for each assay conduction.

Table S5. RT-Q-PCR potency test results obtained during assay validation

Table S5. RT-Q-PCR potency test results obtained during assay validation

Transgene	FcDc (AVE)	SD	CV %
FSP-A1	1.32×10^5	2.60×10^4	19.7
FSP-A2	9.48×10^4	9.91×10^3	10.5
FSP-A3	9.93×10^4	1.27×10^4	12.8
FSP-A4	1.16×10^5	8.62×10^3	7.4

AVE=Average, SD=Standard Deviation, CV=Variation Coefficient. Results are AVE of 3 independent experiments, each including 3 independent infections

Table S6. RT-Q-PCR potency test results obtained on two different GAd-209-FSP clinical lots during assay validation

Table S6. RT-Q-PCR potency test results obtained on two different GAd-209-FSP clinical lots during assay validation

Transgene	Lot #RL18-0010 FcDc (AVE)	Lot #RL22-0026 FcDc (AVE)
FSP-A1	1.32×10^5	1.14×10^5
FSP-A2	9.48×10^4	9.28×10^4
FSP-A3	9.93×10^4	8.22×10^4
FSP-A4	1.16×10^5	1.13×10^5

Results are AVE of 3 independent experiments, each including 3 independent infections

Table S7. Infectious titers under temperature stressed conditions.

Table S7a. Infectious titer for a GAd research batch (RB) after treatment under temperature stressed conditions.

Condition	Infectious titer (ifu/ml)	LRV*
GAd-RB (untreated)	7×10^9	-
GAd-RB (37°C Over-Night)	7.04×10^9	0.002
GAd-RB (37°C 7 days)	2.86×10^9	−0.39
GAd-RB (51°C 1 minute)	5×10^9	−0.15
GAd-RB (51°C 3 minutes)	2.73×10^9	−0,41
GAd-RB (51°C 6 minutes)	3.35×10^8	−1,32

Table S7b. Infectious titer for MVA-209-FSP after treatment under temperature stressed conditions.

Condition	Infectious titer (ifu/ml)	LRV*
MVA-209-FSP (untreated)	1.19×10^8	-
MVA-209-FSP (37°C 24h)	5.03×10^7	−0.37
MVA-209-FSP (37°C 7 days)	9.35×10^6	−1.10
MVA-209-FSP (56°C 10 minutes)	4.53×10^5	−2.42

*LRV = log reduction value

Table S8. Amount of each DS present in GAd-209-FSP DPs and in clinical DP lot #RL22-0026 as quantified by transgene specific Q-PCR

**Table S8.** Amount of each DS present in GAd-209-FSP DPs and in clinical DP lot #RL22-0026 as quantified by transgene specific Q-PCR				
	**DP RL22-0026**	**DP**	**DP-51°C**	**DP-56°C**
GAd-209-FSP-A1 (vp/ml)	6.59×10^{10}	6.39×10^{10}	5.57×10^{10}	5.56×10^{10}
GAd-209-FSP-A2 (vp/ml)	5.76×10^{10}	4.77×10^{10}	4.96×10^{10}	5.11×10^{10}
GAd-209-FSP-A3 (vp/ml)	5.86×10^{10}	5.18×10^{10}	4.63×10^{10}	4.85×10^{10}
GAd-209-FSP-A4 (vp/ml)	6.32×10^{10}	5.41×10^{10}	5.16×10^{10}	5.56×10^{10}
